# Expansion of the ω‐oxidation system AlkBGTL of *Pseudomonas putida* GPo1 with AlkJ and AlkH results in exclusive mono‐esterified dicarboxylic acid production in *E. coli*

**DOI:** 10.1111/1751-7915.12607

**Published:** 2017-03-20

**Authors:** Youri M. van Nuland, Fons A. de Vogel, Gerrit Eggink, Ruud A. Weusthuis

**Affiliations:** ^1^ Bioprocess EngineeringWageningen University and ResearchWageningenthe Netherlands; ^2^ Biobased ProductsWageningen University and ResearchWageningenthe Netherlands

## Abstract

The AlkBGTL proteins coded on the *alk* operon from *Pseudomonas putida* GPo1 can selectively ω‐oxidize ethyl esters of C6 to C10 fatty acids in whole‐cell conversions with *Escherichia coli*. The major product in these conversions is the ω‐alcohol. However, AlkB also has the capacity to overoxidize the substrate to the ω‐aldehyde and ω‐acid. In this study, we show that alcohol dehydrogenase AlkJ and aldehyde dehydrogenase AlkH are able to oxidize ω‐alcohols and ω‐aldehydes of esterified fatty acids respectively. Resting *E. coli* expressing AlkBGTHJL enabled exclusive mono‐ethyl azelate production from ethyl nonanoate, with an initial specific activity of 61 U g_cdw_^−1^. Within 2 h, this strain produced 3.53 mM mono‐ethyl azelate, with a yield of 0.68 mol mol^−1^. This strain also produced mono‐ethyl dicarboxylic acids from ethyl esters of C6 to C10 fatty acids and mono‐methyl azelate from methyl nonanoate. Adding ethyl nonanoate dissolved in carrier solvent bis‐(2‐ethylhexyl) phthalate enabled an increase in product titres to 15.55 mM in two‐liquid phase conversions. These findings indicate that *E. coli* expressing AlkBGTHJL is an effective producer of mono‐esterified dicarboxylic acids from fatty acid esters.

## Introduction

Medium‐chain α,ω‐dicarboxylic acids (DCAs) are building blocks for polyesters, polyurethanes and polyamides. Adipic acid (AA), a C6 DCA, is produced from the petrochemical feedstock benzene. To produce AA, benzene is converted into a mixture of cyclohexanol and cyclohexanone. This mixture is oxidized with nitric acid to yield adipic acid with the concomitant emission of N_2_O, a potent greenhouse gas (Cavani and Alini, 2009; Van de Vyver and Roman‐Leshkov, 2013).

C8‐C10 DCAs are produced from renewable feedstocks, such as oleic acid and ricinoleic acid, *via* ozonolysis (Cornils *et al*., 2000; Metzger, 2009). Although ozonolysis of unsaturated fatty acids is highly selective and effective, it suffers from several drawbacks. The applied ozone is highly toxic and associated with high explosion risks. Furthermore, the process needs a high energy input (Enferadi Kerenkan *et al*., 2016). Finally, the cleavage of unsaturated fatty acids results in by‐product formation.

Direct biocatalytic conversion of medium‐chain fatty acids (MCFAs) and their esters to the corresponding ω‐carboxyl derivative could be a promising alternative production method. Several recent reports demonstrate that the AlkBGT system, part of the *alk* operon from *Pseudomonas putida* GPo1, can ω‐oxidize esterified fatty acids of medium chain length when expressed in *Escherichia coli*. The primary product of the alkane monooxygenase AlkB is the alcohol, but it is also able to oxidize it further to the aldehyde and carboxylic acid, a process called overoxidation. Whole‐cell conversions of nonanoic and dodecanoic methyl esters therefore yield the corresponding ω‐alcohol, ω‐aldehyde and ω‐carboxylic acid (Schrewe *et al*., 2011, 2014; Julsing *et al*., 2012).

**Figure 1:**
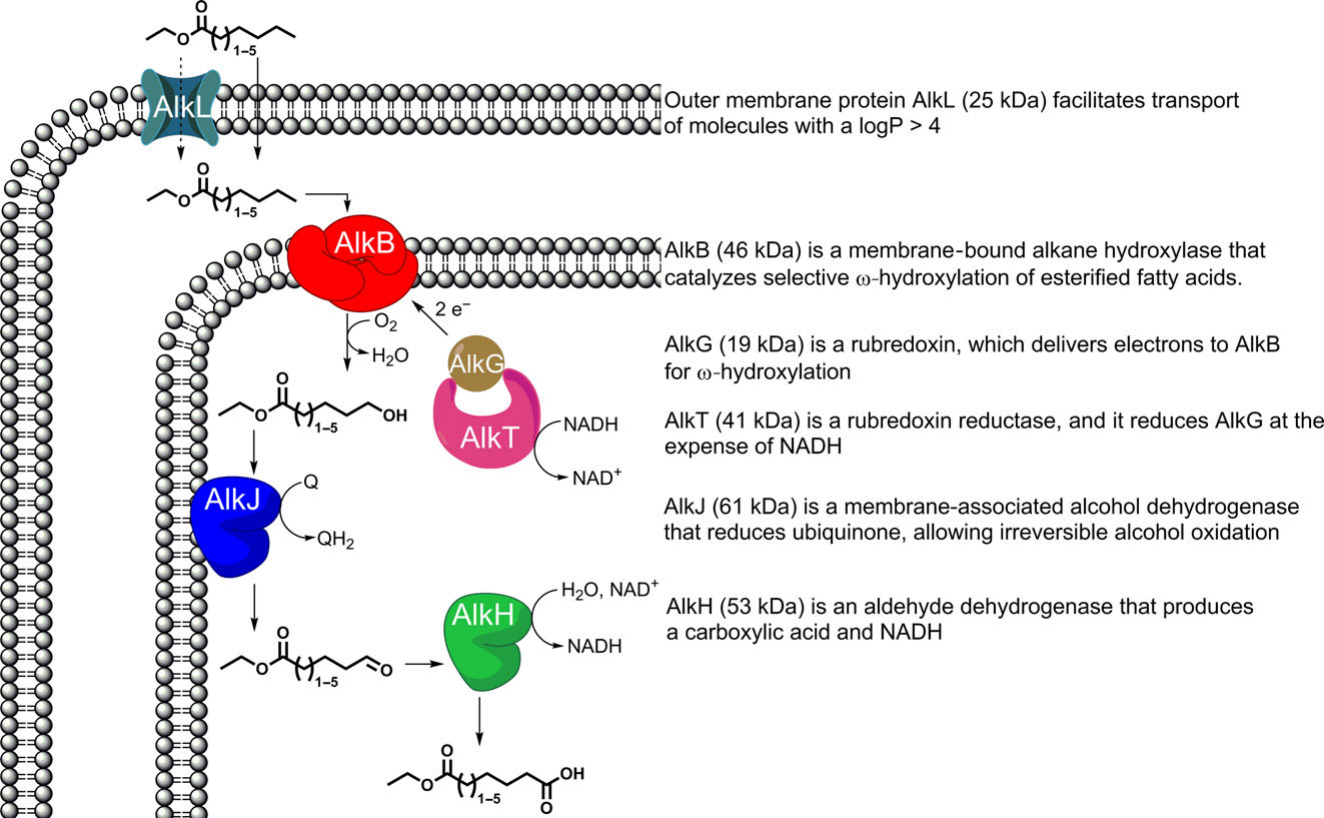
Production of C6 to C10 mono‐ethyl DCAs from ethyl esters, catalysed by AlkBGTHJL.

However, the AlkBGT system is not efficient in the production of carboxylic acids. AlkB prefers esterified fatty acids or alkanes as substrate. This results in high titres of the ω‐alcohol and low conversion rates towards the carboxylic acid. The *alk* operon of *P. putida GPo1* also contains genes encoding alcohol dehydrogenase AlkJ and aldehyde dehydrogenase AlkH (Kok *et al*., 1989; van Beilen *et al*., 1992). Production of carboxylic acids should be more efficient with these enzymes, as the alcohol and aldehyde are natural substrates for AlkJ and AlkH respectively. Furthermore, these enzymes do not require the input of oxygen and NADH, but instead generate reduced cofactors as ubiquinol and NADH (Fig. 1). This would shift the need of NAD^+^ regeneration to regeneration of the ubiquinol. Ubiquinol is regenerated to ubiquinone in the electron transport chain under aerobic conditions.

AlkJ reduces ubiquinone, ensuring irreversible alcohol oxidation (Kirmair and Skerra, 2014). Recently, Schrewe *et al*. (2014) reported that AlkJ converts 12‐hydroxy methyl dodecanoate to 12‐oxo methyl dodecanoate. Expansion of the AlkBGT system with alcohol dehydrogenase AlkJ resulted in a shift of the product distribution towards the aldehyde and acid. Application of the carrier solvent bis(2‐ethylhexyl) phthalate (BEHP) further steered the conversion towards the acid. The products accumulated in the organic phase, showing that they can leave the cell. Bowen *et al*. (2016) have also used AlkJ in combination with a set of aldehyde dehydrogenases in *E. coli*. They applied these enzymes for the production of α,ω‐DCAs, from ω‐hydroxy fatty acids that were produced *de novo* from glucose. AlkJ was clearly the best performer among the tested alcohol dehydrogenases.

For further oxidation of the ω‐aldehyde of alkyl esters, aldehyde dehydrogenase AlkH is a promising candidate as it converts medium‐chain aldehydes to carboxylic acids. AlkH has been applied as part of the *alk* operon for conversion of alkanes to fatty acids (Favre‐Bulle and Witholt, 1992; Grant *et al*., 2011), but not for ω‐oxidized alkyl esters.

Recently, we have demonstrated that *E. coli* expressing AlkBGT(L) can also ω‐oxidize esters with an alkyl chain > 1 (van Nuland *et al*., 2016). By increasing the alkyl chain length, the ω‐oxidation activity on shorter fatty acid esters improved greatly. Outer membrane protein AlkL was beneficial when the sum of the alkyl and acyl chain length exceeded 10. In these conversions, the ω‐alcohol was also the major product. The ratio of ω‐alcohol:ω‐acid was 100:17, and the ω‐aldehyde was only detected in trace amounts. Alcohol dehydrogenase and aldehyde dehydrogenase activities are required to fully convert esterified fatty acids in mono‐esterified dicarboxylic acids. The combined action of AlkJ and AlkH seems appropriate for this conversion, but has never been tested with ethyl‐esterified fatty acids before. Our aim was to investigate whether AlkJ and AlkH can improve production of the ω‐carboxylic acid of ethyl‐esterified fatty acids, and whether application of carrier solvent BEHP can enhance production.

## Results

As AlkB can overoxidize ω‐alcohols, it can impede the assessment of the performances of AlkJ and AlkH in the AlkBGTHJL pathway. Therefore, we decided to test AlkJ and AlkH individually. This should allow us to compare AlkJ and AlkH activities with the activities of AlkBGT on the intermediates of the pathway. Conversion tests were carried out with resting *E. coli* cells, expressing these proteins. Activities of these cells are expressed in U g_cdw_^−1^, where 1 U equals 1 μmol product formed per minute.

### Testing functionality of AlkJ

**Figure 2:**
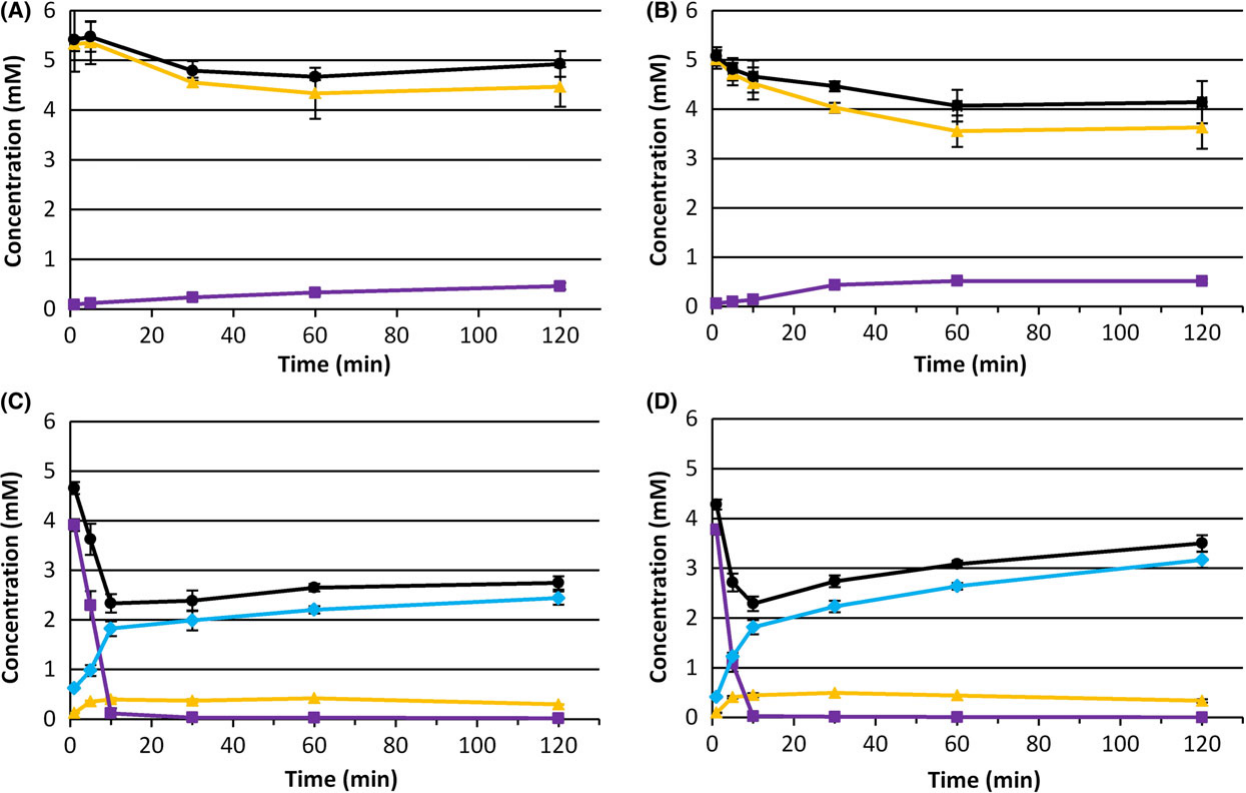
Resting‐cell conversions of 5 mM 9‐hydroxy ethyl nonanoate (panel A and B) or 9‐oxo methyl nonanoate (panel C and D) at 37°C. Panel A: *E. coli* pCOM10_*alkJ*, 1.0 gcdw l^−1^. Panel B: *E. coli* pCOM10_*alkJL*, 1.0 gcdw l^−1^. Panel C: *E. coli* pCOM10_*alkH*, 1.1 gcdw l^−1^. Panel D: *E. coli* pCOM10_*alkHL*, 1.1 gcdw l^−1^. Triangles: ω‐alcohol. Squares: ω‐aldehyde. Diamonds: ω‐acid. Circles: sum.

We checked whether AlkJ was functionally expressed in *E. coli* NEBT7 by testing the oxidation of 9‐hydroxy ethyl nonanoate. Resting cells of *E. coli* pCOM10*_alkJ* and *E. coli* pCOM10_*alkJL* were incubated with 9‐hydroxy ethyl nonanoate (see Fig. 2A and B).

Both strains produced 9‐oxo ethyl nonanoate, indicating that AlkJ was functionally expressed and uses 9‐hydroxy ethyl nonanoate as substrate. Coexpression of AlkJ and AlkL from pCOM10_*alkJL* resulted in lower activities (see Table 1), but the product titres reached after 2 h were similar (0.46 versus 0.51 mM). The conversion was incomplete; only 10% of the substrate was used, suggesting that an equilibrium was reached or that the enzyme is unstable.

**Table 1:** Initial (1 min) activities (U g_cdw_^−1^) of various resting *E. coli* NEBT7 strains in the presence of 5 mM 9‐hydroxy ethyl nonanoate (9HNAEE), 9‐oxo methyl nonanoate (9ONAME) or ethyl nonanoate (NAEE). Negative values indicate the reverse reaction

Strain	Substrate	T (°C)	−CH_3_ →−CH_2_OH	Reaction	
				−CH_2_OH → −CH=O	−CH=O → −COOH
pCOM10_*alkL*	9HNAEE	30	ND	ND	ND
	9ONAME	30	ND	−62 ± 1	14 ± 4
pBTL10	9HNAEE	30	ND	24 ± 0	ND
		37	ND	30 ± 1	27 ± 8
	9ONAME	30	ND	−78 ± 8	200 ± 17
		37	ND	−31 ± 3	101 ± 6
	NAEE	30	51 ± 1	ND	ND
pCOM10_*alkJ*	9HNAEE	30	ND	102 ± 6	ND
		37	ND	76 ± 8	ND
pCOM10_*alkJL*	9HNAEE	30	ND	254 ± 4	ND
		37	ND	61 ± 2	ND
pCOM10_*alkH*	9ONAME	30	ND	−102 ± 12	257 ± 16
		37	ND	−116 ± 22	594 ± 12
pCOM10_*alkHL*	9ONAME	30	ND	−88 ± 8	380 ± 20
		37	ND	−88 ± 7	383 ± 17
pBGTHJL	9HNAEE	30	ND	227 ± 10	58 ± 11
	9ONAME	30	ND	−121 ± 11	696 ± 72
	NAEE	30	81 ± 4	61 ± 3	61 ± 3

ND, not detected.

We performed the same conversions at 30°C, to see if this would improve the activity. This temperature corresponds to the optimal growth temperature of *Pseudomonas putida* GPo1. *E. coli* pCOM10_*alkJ* performed somewhat better at 30°C regarding initial activity (Table 1). The final product titres reached were lower (0.28 versus 0.46 mM; see Fig. S1). *E. coli* pCOM10_*alkJL*, however, showed about fourfold higher initial activity at 30°C. Final product titres were also slightly higher at 30°C. A control experiment was carried out at 30°C with *E. coli* pCOM10_*alkL. *This test confirmed that the production of 9‐oxo ethyl nonanoate was due to the presence of AlkJ, as no 9‐oxo ethyl nonanoate was produced by the pCOM10_*alkL* strain (Table 1; Fig. S4).

### Testing functionality of AlkH

With the same approach, we checked whether AlkH could be functionally expressed in *E. coli* NEBT7 at 37°C. The substrate we used for these tests was 9‐oxo methyl nonanoate, as 9‐oxo ethyl nonanoate was not commercially available. *E. coli* pCOM10_*alkH* and *E. coli* pCOM10_*alkHL* catalysed both the reduction and oxidation of the substrate, but the oxidation activity was much higher (Fig. 2C and D). At 30°C, the oxidation activity of *E. coli* pCOM10_*alkH* was lower, but final product concentrations were similar (Table 1 and Fig. S2). For *E. coli* pCOM10_*alkHL*, there were no differences between the two tested temperatures.

The carbon balances of these tests were not complete. We did not detect a by‐product in these tests. The same experiment was carried out with *E. coli* pCOM10_*alkL* at 30°C (see Table 1). This strain mainly reduced the substrate to the ω‐alcohol. This indicates that a native *E. coli* enzyme was responsible for this reaction, also in strain pCOM10_*alkH* and pCOM10_*alkHL*. With these strains, a similar gap in the C‐balance appeared. Volatility cannot be the cause of this gap, because no decrease in the concentration of 9‐oxo methyl nonanoate occurred during abiotic incubations in resting‐cell buffer (Fig. S5). Also, the gap became smaller towards the end of the incubation, suggesting that the substrate was released again. These findings suggest that the substrate reversibly binds to the *E. coli* biomass.

### Testing AlkB overoxidation capacity

To evaluate whether AlkJ/AlkH can contribute more to ω‐acid production than AlkB, it is necessary to determine the overoxidation capacity of AlkB. AlkB overoxidizes ethyl nonanoate to 9‐oxo ethyl nonanoate and mono‐ethyl azelate (van Nuland *et al*., 2016). We determined this overoxidation activity of whole cells expressing AlkBGTL from the pBTL10 plasmid by the addition of the intermediates 9‐hydroxy ethyl nonanoate and 9‐oxo methyl nonanoate and measuring the initial activities (Table 1). *E. coli* pBTL10 oxidized 9‐hydroxy ethyl nonanoate to 9‐oxo ethyl nonanoate, with an initial activity of 24 U g_cdw_^−1^ at 30°C. The activity of *E. coli* pCOM10_*alkJL* was about 10‐fold higher (254 U g_cdw_^−1^). Further oxidation to mono‐ethyl azelate was only detected after 5 min; at 37°C, this activity was higher. *E. coli* pBTL10 converted the aldehyde at much higher rates, to both the alcohol (78 U g_cdw_^−1^) and mono‐methyl azelate (200 U g_cdw_^−1^); at 37°C, those rates were lower. Still, *E. coli* pCOM10_*alkHL* produced mono‐methyl azelate with a higher activity (380 U g_cdw_^−1^).

### Ethyl ester conversion with *E. coli* pBGTHJL

We then proceeded with whole‐cell conversions using *E. coli* that expressed besides AlkBGTL, also AlkJ and AlkH from the pBGTHJL plasmid. Tests with either 9‐hydroxy ethyl nonanoate or 9‐oxo methyl nonanoate were carried out, to verify functionality of AlkJ and AlkH when expressed as part of the AlkBGTHJL pathway. *E. coli* pBGTHJL oxidized 9‐hydroxy ethyl nonanoate to 9‐oxo ethyl nonanoate with an initial activity of 227 U g_cdw_^−1^, which was nearly 10 times higher than the activity of *E. coli* pBTL10 (Table 1). The pBGTHJL strains oxidized 9‐oxo ethyl nonanoate at 696 U g_cdw_^−1^; this activity was much lower with *E. coli* pBTL10 (Table 1). Both AlkJ and AlkH were thus functioning when expressed from the pBGTHJL plasmid.

**Figure 3:**
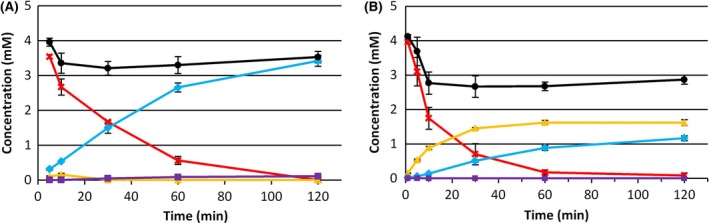
Whole‐cell conversion of 5 mM ethyl nonanoate by *E. coli* pBGTHJL at 30°C with 1.1 g_cdw_ l^−1^ biomass (A) and 37°C with 1.1 g_cdw_ l^−1^ biomass (B). Crosses: ethyl nonanoate. Triangles: ω‐alcohol. Squares: ω‐aldehyde. Diamonds: ω‐acid. Circles: sum.

Subsequently, we did conversions with ethyl nonanoate, using *E. coli* pBGTHJL (Fig. 3). Conversions at 37°C pointed out that a large share of 9‐hydroxy ethyl nonanoate was not converted to mono‐ethyl azelate (alcohol:acid ratio 1:0.72). At 30°C however, no 9‐hydroxy ethyl nonanoate was detected anymore after 2 h. Conversions of 5 mM ethyl nonanoate yielded 3.42 mM of mono‐ethyl azelate, corresponding to a yield of 0.68 mol mol^−1^. The intermediate products 9‐hydroxy ethyl nonanoate and 9‐oxo ethyl nonanoate did not accumulate above 0.15 mM. Moreover, at 2 h hardly any intermediate product accumulated. It is noteworthy to mention that the ester group was only hydrolysed to a very limited extent (0.07 mM after 2 h).

After 2 h of incubation, the sum of products and substrate was 3.53 mM, 71% of the 5 mM substrate that was added. The rapid decrease in substrate in the first minutes of the conversion and the fact that ethyl nonanoate is volatile suggest that evaporation caused this gap. We set up a negative control experiment to test whether evaporation was the cause of this loss. The same set‐up was applied, using 1.0 g_cdw_ l^−1^ *E. coli* pCOM10_*alkL*, a strain that is unable to ω‐oxidize ethyl nonanoate. After 5 min of incubation, 52% of the added ethyl nonanoate was left, and after 2 h, only 23% (Fig. S3). Ester hydrolysis occurred, but only 0.07 mM nonanoic acid was detected after 2 h. Evaporation thus caused the gap between added substrate and the sum of products and substrate after the conversion.

### Substrate specificity

Ethyl esters of C6 to C10 fatty acids were also tested as substrate (Table 2). From all substrates, the pBGTHJL strain produced the corresponding mono‐ethyl dicarboxylic acid. Also methyl nonanoate was tested, because the activity of AlkB on this substrate is the highest reported in the literature (Julsing *et al*., 2012). The titre of mono‐methyl azelate was comparable to the titre of mono‐ethyl azelate. Product titres from other chain lengths were considerably lower than from methyl nonanoate and ethyl nonanoate. In samples with ethyl heptanoate and ethyl octanoate, a large share of the added substrate was still present after 2 h. Conversions with ethyl heptanoate yielded clearly less product compared to the other substrates. Also the alcohol accumulated when ethyl esters of shorter fatty acids were used as substrate.

**Table 2:** Conversion of 5 mM C6 to C10 ethyl esters by *E. coli* pBGTHJL. Reactions were incubated for 2 h at 30°C, with 1.1 g_cdw_ l^−1^ biomass

Substrate	Concentration (mM)				
	ω‐Alcohol	ω‐Aldehyde	ω‐Acid	Substrate	Sum
Ethyl hexanoate^a^	1.00 ± 0.01	ND	1.39 ± 0.09	0.33 ± 0.01	2.72 ± 0.10
Ethyl heptanoate	0.24 ± 0.01	0.13 ± 0.01	0.17 ± 0.00	2.65 ± 0.03	3.19 ± 0.03
Ethyl octanoate	1.55 ± 0.27	ND	1.00 ± 0.36	1.69 ± 0.92	4.23 ± 0.80
Ethyl nonanoate	ND	0.07 ± 0.01	3.46 ± 0.14	0.13 ± 0.03	3.66 ± 0.10
Ethyl decanoate	ND	ND	1.49 ± 0.10	0.18 ± 0.04	1.67 ± 0.07
Methyl nonanoate^a^	ND	ND	3.45 ± 0.46	0.13 ± 0.06	3.58 ± 0.52

ND, not detected. ^a^Tests were carried out in duplicate.

### Application of organic phase

For mono‐ethyl azelate production, substrate concentrations appeared to be limiting after 2 h (Fig. 3). The addition of 10 mM ethyl nonanoate instead of 5 mM ethyl nonanoate did not improve product titres (data not shown). The biocompatible organic solvent bis‐(2‐ethylhexyl) phthalate (BEHP) has been applied before to act as a substrate reservoir and product sink for similar bioconversions (Schrewe *et al*., 2014). We tested whether a two‐liquid phase approach with BEHP as organic phase could enhance product yields. Ethyl nonanoate was tested as substrate and was added as a 25% (v/v) solution in BEHP (Table 3).

**Table 3:** Resting *E. coli* pBGTHJL conversions of ethyl nonanoate in a two‐liquid phase set‐up, with 25% ethyl nonanoate in BEHP as organic phase. 9HNAEE: 9‐hydroxy ethyl nonanoate; MEA: mono‐ethyl azelate; AzA: azelaic acid. The applied biomass concentration was 1.0 g_cdw_ l^−1^

	Product concentration (mM)						% Conversion
	9HNAEE		MEA		AzA		
Incubation time (h)	Aqueous	Organic	Aqueous	Organic	Aqueous	Organic	
2	ND	ND	1.35 ± 0.13	1.09 ± 0.34	0.10 ± 0.04	ND	0.22
18	0.04 ± 0	2.12 ± 0.23	1.63 ± 0.08	15.55 ± 1.09	0.14 ± 0.02	1.12 ± 0.27	1.78

ND, not detected.

In this set‐up, mono‐ethyl azelate was formed. These results indicate that *E. coli* pBGTHJL can efficiently convert the alcohol and aldehyde to the acid. Besides mono‐ethyl azelate, also low amounts of azelaic acid were formed, indicating that *E. coli* pBGTHJL is able to hydrolyse the ester bond to a limited extent. After 2 h of incubation, this set‐up resulted in formation of 2.54 mmol of product per litre of aqueous medium, less than the 3.53 mmol when the substrate was directly added. After 18 h however, the formed product amounted to 20.60 mmol (1.78% of substrate converted), and the majority of the products were carboxylic acids. Most of these acids were detected in the organic phase. The addition of ethyl nonanoate dissolved in BEHP thus decreased the productivity, but enabled higher final product titres. There was also a clear difference in the distribution of mono‐ethyl azelate over the two phases at the two time points. At 2 h, the ratio organic:aqueous was 0.81:1, and at 18 h, this shifted to 9.54:1, meaning that at a later stage mono‐ethyl azelate partitioned better in the organic phase. This was most likely due to a decrease in the pH of the aqueous medium, resulting in a higher concentration of protonated mono‐ethyl azelate. This allowed accumulation of the acid in BEHP.

## Discussion

AlkJ was applied before to catalyse the conversion of alcohols to aldehydes in *E. coli* (Schrewe *et al*., 2014; Song *et al*., 2014; Bowen *et al*., 2016). This is the first report of its functionality on 9‐hydroxy ethyl nonanoate. Schrewe and colleagues reported that *E. coli* W3110 expressing AlkJ converted 12‐hydroxy methyl dodecanoate to 12‐oxo methyl dodecanoate, with an initial whole‐cell activity of 78.9 U g_cdw_^−1^ (measured at 5 min). The activity of *E. coli* NEBT7‐pCOM10*_alkJ* on 9‐hydroxy ethyl nonanoate we report here is 102 U g_cdw_^−1^ (measured at 1 min), which is higher but in the same order of magnitude. The final product titres were lower; the system seems to reach equilibrium already at low titres of the aldehyde. We noticed that pCOM10‐AlkL showed native 9‐oxo methyl nonanoate activity, and apparently this activity interfered in the AlkJ assay. Initial activities of AlkJ were higher at 30°C. AlkJ was shown before to have only 50% residual activity at 34°C *in vitro* (Kirmair and Skerra, 2014), suggesting that incubation at 37°C greatly decreases the stability of AlkJ.

AlkH has only been applied before as part of the *alk* operon or modified versions of that operon, to our knowledge. Here, we have confirmed the aldehyde dehydrogenase activity of this protein, by the addition of 9‐oxo methyl nonanoate to resting cells expressing AlkH. Among the tested strains in this study, *E. coli* expressing AlkH clearly shows the highest activity (257, 594 U g_cdw_^−1^ when coexpressed with AlkL).

Coexpression of AlkJ and AlkH with AlkL improved whole‐cell activities at 30°C, but at 37°C the activity actually decreased. Potentially, overexpression of AlkL at 37°C causes misfolding of the protein. It has been shown before that high expression levels of AlkL can negatively affect biocatalyst performance (Grant *et al*., 2014).

We compared the activities of AlkJ and AlkH with the overoxidation activity of AlkB. Both the activities of AlkJ and AlkH were higher than activities of AlkB. Compared to results from similar tests with methyl dodecanoate and the corresponding ω‐oxidized derivatives reported by Schrewe *et al*. (2014), there were some clear differences. Firstly, in the study by Schrewe *et al*., *E. coli* expressing AlkB oxidized 12‐hydroxy methyl dodecanoate and 12‐oxo methyl dodecanoate roughly at the same rate. Here, we describe that AlkB displays quite different activities on the alcohol and aldehyde of C9‐esters. An activity of only 24 U g_cdw_^−1^ was reported with 9‐hydroxy ethyl nonanoate as substrate. Potentially this compound becomes too polar for the active site of AlkB due to the fact that the molecule is two carbon atoms shorter. The rate with 9‐oxo methyl nonanoate was much higher, 200 U g_cdw_^−1^. This could be due to the fact that a methyl ester was the substrate. AlkB activities are higher on methyl nonanoate then on ethyl nonanoate (Julsing *et al*., 2012; van Nuland *et al*., 2016); this might apply to the ω‐aldehydes as well. Still, this would not explain the large differences between the rates of alcohol and aldehyde oxidation.

Secondly, the reduction of 9‐oxo methyl nonanoate occurred at high rates. Host intrinsic reduction of 12‐oxo methyl dodecanoate has been reported by Schrewe *et al*., but at a significantly lower activity (~6 U g_cdw_^−1^), indicating that this activity is much higher for shorter chain lengths. This activity is most likely caused by an alcohol dehydrogenase, resulting in oxidation of NAD(P)H. This can result in a futile cycle, with a loss of energy as a consequence. We also observed the reduction of 9‐oxo methyl nonanoate by *E. coli* pBGTHJL, when 5 mM 9‐oxo methyl nonanoate was added as substrate. However, in tests where ethyl nonanoate was the substrate, both 9‐hydroxy ethyl nonanoate and 9‐oxo ethyl nonanoate concentrations remained low. This suggests that AlkH outcompetes the reductive activity under these conditions. To prove this however, more kinetic information of the involved enzymes would be necessary.

Because of the high activities of AlkJ and AlkH on ω‐oxidized esters, we expected that a pathway consisting of AlkBGTHJL would outperform the AlkBGTL pathway concerning the yield of mono‐ethyl DCA. Pathway AlkBGTHJL facilitated exclusive mono‐ethyl DCA production from ethyl nonanoate and methyl nonanoate. This exclusive production was a clear improvement compared to earlier studies, wherein AlkBGTL or AlkBGTJL enzymes were applied (Schrewe *et al*., 2011, 2014; Julsing *et al*., 2012; van Nuland *et al*., 2016). Furthermore, mono‐ethyl dicarboxylic acid production using AlkBGTHJL also requires less energy and oxygen input than when only AlkBGTL is applied. Complete oxidation to the acid *via* AlkB requires 3 NADH and 3 mol O_2_, whereas with AlkBGTHJL this would yield a reduced ubiquinone (assuming 100% coupling efficiency) and only require 1 mol O_2_ for the ω‐oxidation pathway (Schrewe *et al*., 2014) (see Fig. S6). The addition of AlkJ and AlkH also shifts the need of NAD^+^ recycling to ubiquinol recycling. This increases the need of O_2_ by 0.5 mol to 1.5 mol O_2_ in total and delivers ATP.

AlkH also accepts 9‐oxo ethyl nonanoate, because high titres of mono‐ethyl azelate were reached and there was no 9‐oxo ethyl nonanoate accumulation. Hence, this pathway is a promising biocatalytic route for medium‐chain α,ω‐bifunctional monomers. The initial specific activity in the presence of glucose was 81 U g_cdw_^−1^, which is somewhat higher than the 70 U g_cdw_^−1^ reported for ω‐oxidation of ethyl nonanoate by *E. coli* expressing AlkBGTL at 37°C (van Nuland *et al*., 2016). The presence of AlkJ and AlkH caused low alcohol concentrations, which may have resulted in less competition for the active site of AlkB and thus allowed more ethyl nonanoate to be converted. The aldehyde concentration never exceeded 0.1 mM. This can be explained from the high activity of AlkH and to a lesser extent AlkB on this intermediate.

Hydrolysis of the ethyl ester bond only occurred to a limited extent in the tests without organic phase. The predominant product in these conversions is thus a mono‐ester. This is not the case with *Candida tropicalis*, an industrially relevant dicarboxylic acid producer, because this strain hydrolyses the ester bond (Picataggio *et al*., 1992; Lu *et al*., 2010). Mono‐esters can be useful starting materials for synthetic chemistry routes, such as high‐yield di‐ester production *via* Kolbe electrolysis (Schäfer, 2012).

This pathway also accepted other chain lengths from C6 to C10. Hence, also the industrially relevant mono‐ethyl adipate and mono‐ethyl sebacate were formed. These could serve as precursors for the corresponding di‐acids or di‐esters. The product titres were lower with chain lengths other than C9. In the cases of ethyl hexanoate and ethyl octanoate, a large share of the products was the alcohol. Either the alcohol/aldehyde dehydrogenases are not active enough on these substrates, or *E. coli* NEBT7 has such high aldehyde reduction activity that it causes the alcohol to accumulate. If this is the case, knocking out the enzyme responsible for this reduction would be a necessity, although this enzyme can be essential. It must be noted that especially the ethyl esters of shorter fatty acids are volatile, which affects the obtained yields. Ethyl heptanoate does not seem to be a good substrate, as can be concluded from the low product titres. Methyl/ethyl heptanoate were tested before as substrates for the AlkBGT system and were also not ω‐oxidized efficiently (Schrewe *et al*., 2011; van Nuland *et al*., 2016). The reason for this remains unknown.

When we applied BEHP containing a high concentration of ethyl nonanoate, the productivity declined. This lower productivity could have been a result of mass transfer limitation of ethyl nonanoate to the aqueous phase. Moreover, the conversions took place in a rotary shaker, which likely resulted in suboptimal mixing. Nevertheless, this set‐up enabled much higher mono‐ethyl azelate concentrations, with a low biomass concentration. The observed accumulation of mono‐ethyl azelate in BEHP could facilitate downstream processing. *E. coli* NEBT7 has some esterase activity, as azelaic acid was accumulating in these tests. This phenomenon was observed before with *E. coli* W3110 pBTL10/pBTLJ10 with methyl dodecanoate as substrate (Schrewe *et al*., 2014).

## Conclusion

The expansion of the AlkBGTL pathway with AlkJ and AlkH in *E. coli* resulted in a biocatalyst that could efficiently ω‐oxidize ethyl‐esterified medium‐chain fatty acids to yield mono‐ethyl DCAs. Alcohol dehydrogenase AlkJ produces the aldehyde from 9‐hydroxy ethyl nonanoate. Aldehyde dehydrogenase AlkH produces the carboxylic acid from 9‐oxo methyl nonanoate. These enzymes enable *E. coli* to completely convert products from AlkBGTL to the carboxylic acid, when methyl or ethyl nonanoate is used as substrate. *E. coli* expressing AlkBGTHJL is thus a more efficient producer of mono‐esterified DCAs compared to *E. coli* expressing AlkBGT or AlkBGTJL.

Mono‐ethyl DCA production was also possible with other chain lengths, ranging from C6 to C10. This highlights the broad applicability of the AlkBGTHJL pathway for (mono‐ethyl) DCA production.

The addition of ethyl nonanoate as a 25% solution in carrier solvent BEHP boosted the production of carboxylic acids and can act as a biocompatible substrate reservoir.

This work demonstrates the possibility of producing (mono‐esterified) DCAs from medium‐chain esterified fatty acids directly. Application of whole cells for multistep biocatalysis of chemically challenging reactions holds great promise. These findings could eventually lead to more sustainable production of industrially relevant DCAs.

## Experimental procedures

### Plasmids, strains and chemicals

The plasmids used in this study are listed in Table 4. *E. coli* TOP10 (Invitrogen™, Carlsbad, CA, USA) was used for cloning purposes. *E. coli*‐NEBT7 (New England Biolabs™, Ipswich, MA, USA) was used for conversion studies.

**Table 4:** Plasmids used in this study

Plasmids	Characteristics	References
pCOM10_*alkL*	*alkL* on pCOM10	Julsing *et al*. (2012)
pGEc47	*alk* operon on pLAFR1	Eggink *et al*. (1987)
pSTL	*alkTL* on pCOM10	van Nuland *et al*. (2016)
pCOM10_*alkJ*	*alkJ* on pCOM10	This study
pCOM10_*alkJL*	*alkJL* on pCOM10	This study
pCOM10_*alkH*	*alkH* on pCOM10	This study
pCOM10_*alkHL*	*alkHL* on pCOM10	This study
pBTL10	*alkBGTL* on pCOM10	van Nuland *et al*. (2016)
pBGTHJL	*alkBGTHJL* on pCOM10	This study

### Construction of plasmids

For construction of pSJ and pSH vectors, *alkJ* and *alkH* were amplified from pGEc47. These were ligated in digested pCOM10_*alkL* (digestion resulted in removal of *alkL*). *alkL* was then ligated into the digested pSJ and pSH vectors, to yield pSJL and pSHL.

pSTBGHJL was constructed as follows. The *alkBFGHJ* and *alkL* fragments were amplified from pGEc47, and the primers were designed in such a way that *alkL* ligated to a 3' overhang of the *alkBFGHJ* fragment. This fragment was ligated into a digested pSTL plasmid, to yield pSTBFGHJL.

### Chemicals

Chemicals were ordered with the highest purity available from the following vendors:

Sigma‐Aldrich (St Louis, MO, USA): dodecane, tetradecane, ethyl hexanoate, ethyl nonanoate, 6‐hydroxy ethyl hexanoate, 7‐hydroxy ethyl heptanoate. Alfa Aesar: ethyl octanoate, ethyl decanoate, bis(2‐ethylhexyl) phthalate, adipic acid mono‐ethyl ester, pimelic acid mono‐ethyl ester, suberic acid mono‐ethyl ester, sebacic acid mono‐ethyl ester. TRC (North York, ON, Canada): 9‐hydroxy nonanoic acid ethyl ester, 9‐oxo methyl nonanoate, azelaic acid mono‐ethyl ester. Merck(Darmstadt, Germany): ethyl heptanoate.

### Cultivation and bioconversions

Cultivation and bioconversions were carried out as described before (van Nuland *et al*., 2016), except the temperature, which was set to 30°C for most experiments.

Conversions with organic phase were carried out similarly, except that a solution of 25% ethyl nonanoate in bis‐2‐ethylhexyl phthalate was added to the reaction, in a 1:1 ratio. Furthermore, ethanol was not added. For sampling, equal volumes of the water phase and organic phase were withdrawn.

### GC analysis

Aqueous samples were treated with 1% of an 85% phosphoric acid solution and then extracted with CHCl_3_ containing 0.2 mM dodecane or tetradecane as an internal standard. Organic phase samples were diluted 40 times in CHCl_3_ containing 0.2 mM tetradecane. Samples were analysed with and without derivatization with TMSH. For qualitative analysis, samples were analysed with a Thermo Scientific TRACE Ultra gas chromatograph coupled to a DSQII mass spectrometer. Quantitative analysis was carried out with an Agilent 6890 or 7890 GC coupled to an FID. Response factors of chemicals that were not commercially available were based on structurally similar chemicals.

## Conflict of interest

None declared.

## Supporting information

Fig. S1. Resting cell conversions of 9‐hydroxy ethyl nonanoate at 30°C.
Fig. S2. Resting cell conversions of 9‐oxo methyl nonanoate at 30°C.
Fig. S3. Incubation of ethyl nonanoate with 1.0 g_cdw_/L of *E. coli* pCOM10_*alkL* (solid line), and without cells (dashed line).
Fig. S4. Incubation of 9‐hydroxy ethyl nonanoate with 1.0 g_cdw_/L of *E. coli* pCOM10_*alkL* (solid line), and without cells (dashed line).
Fig. S5. Incubation of 9‐oxo methyl nonanoate with 1.0 g_cdw_/L of *E. coli* pCOM10_*alkL* (solid lines), and without cells (dashed line).
Fig. S6. Comparison of the AlkBGTHJ pathway with the AlkBGT (overoxidation) pathway, for ω‐oxidation of ethyl esterified fatty acids.
