# Supplementary material for: Expansion of the ω‐oxidation system AlkBGTL of Pseudomonas putida GPo1 with AlkJ and AlkH results in exclusive mono‐esterified dicarboxylic acid production in E. coli
Source: Microb Biotechnol. 2017 Mar 20;10(3):594–603. doi: 10.1111/1751-7915.12607 (PMC5404194; doi:10.1111/1751-7915.12607)
Supplement: Supplementary file 1 — Fig. S1. Resting cell conversions of 9‐hydroxy ethyl nonanoate at 30°C. Fig. S2. Resting cell conversions of 9‐oxo methyl nonanoate at 30°C. Fig. S3. Incubation of ethyl nonanoate with 1.0 gcdw/L of E. coli pCOM10_alkL (solid line), and without cells (dashed line). Fig. S4. Incubation of 9‐hydroxy ethyl nonanoate with 1.0 gcdw/L of E. coli pCOM10_alkL (solid line), and without cells (dashed line). Fig. S5. Incubation of 9‐oxo methyl nonanoate with 1.0 gcdw/L of E. coli pCOM10_alkL (solid lines), and without cells (dashed line). Fig. S6. Comparison of the AlkBGTHJ pathway with the AlkBGT (overoxidation) pathway, for ω‐oxidation of ethyl esterified fatty acids. [file MBT2-10-594-s001.docx]

Supplementary material to ‘Expansion of the omega oxidation system AlkBGTL of *Pseudomonas putida* GPo1 with AlkJ and AlkH results in exclusive mono-esterified dicarboxylic acid production in *E. coli*’

#
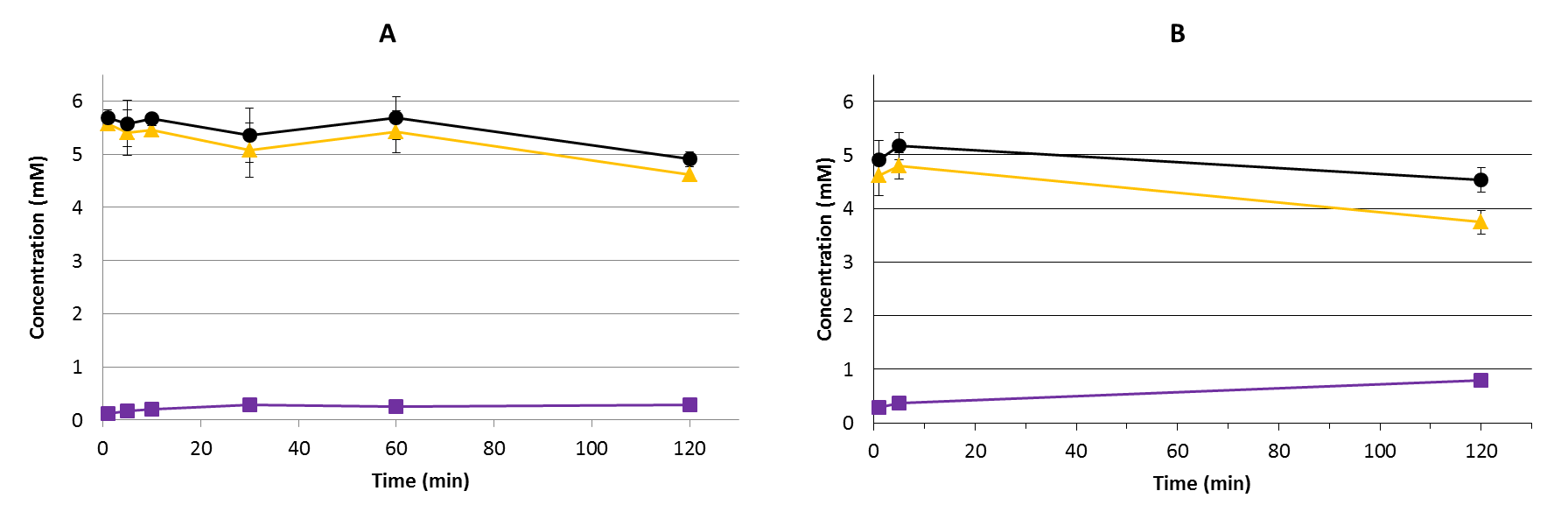


Figure S1. Resting cell conversions of 9-hydroxy ethyl nonanoate at 30 °C. Panel A: *E. coli* pCOM10_*alkJ*, 1.1 g_cdw_/L. Panel B, *E. coli* pCOM10_*alkJL* 1.1 g_cdw_/L. Squares: 9-oxo ethyl nonanoate. Triangles: 9-hydroxy ethyl nonanoate. Circles: sum.


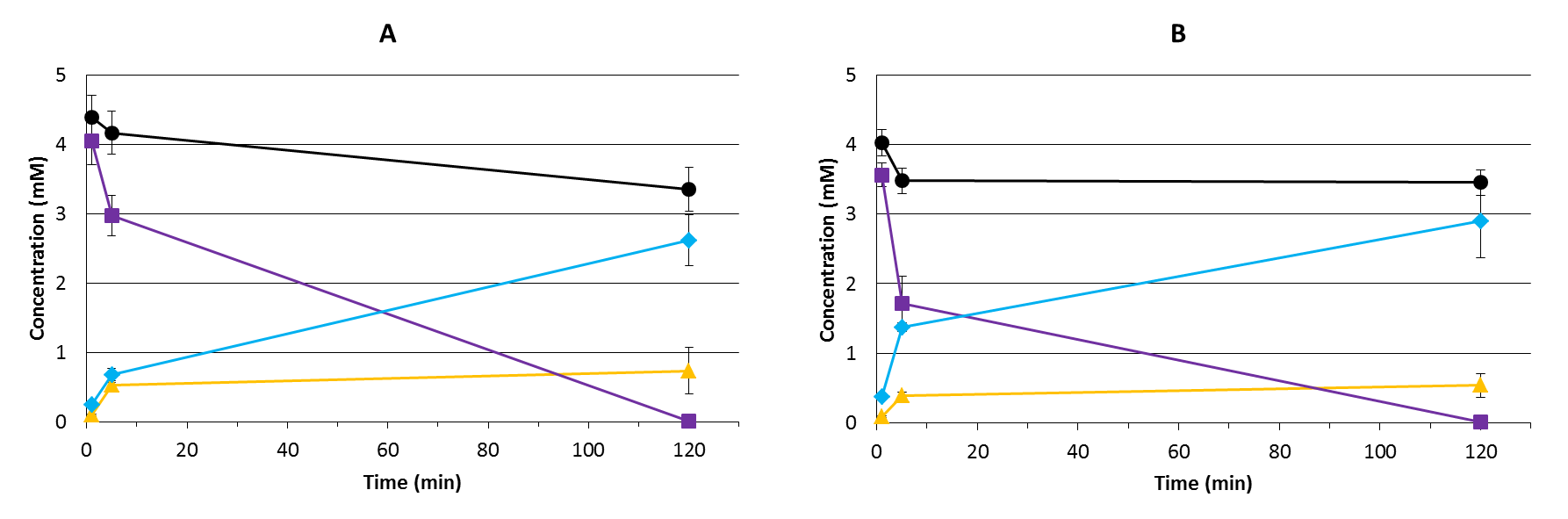


Figure S2. Resting cell conversions of 9-oxo methyl nonanoate at 30 °C. Panel A: *E. coli* pCOM10_*alkH*, 1.0 g_cdw_/L. Panel B, *E. coli* pCOM10_*alkHL* 1.0 g_cdw_/L. Squares: 9-oxo methyl nonanoate. Triangles: 9-hydroxy methyl nonanoate. Diamonds: mono-methyl azelate. Circles: sum.


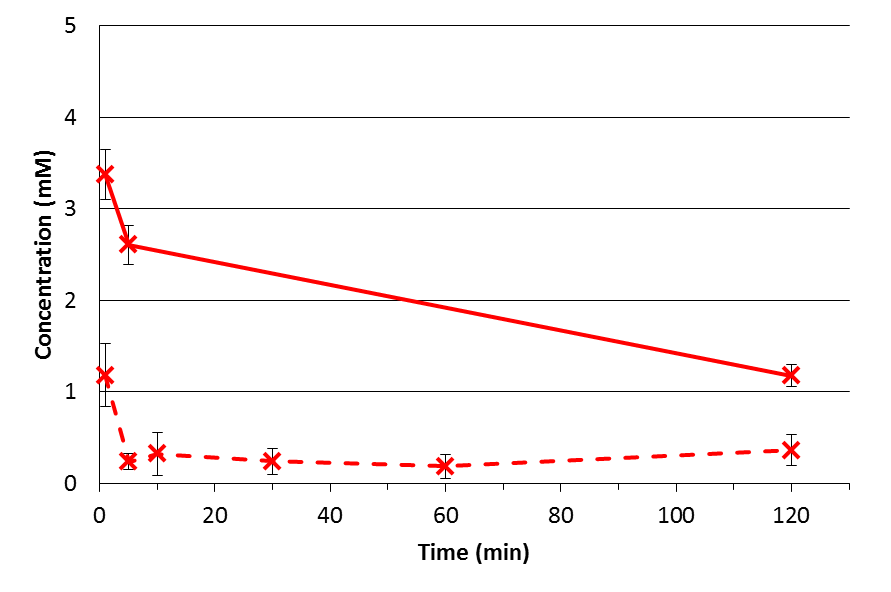


Figure S3. Incubation of ethyl nonanoate with 1.0 g_cdw_/L of *E. coli* pCOM10_*alkL* (solid line), and without cells (dashed line). Crosses: ethyl nonanoate.


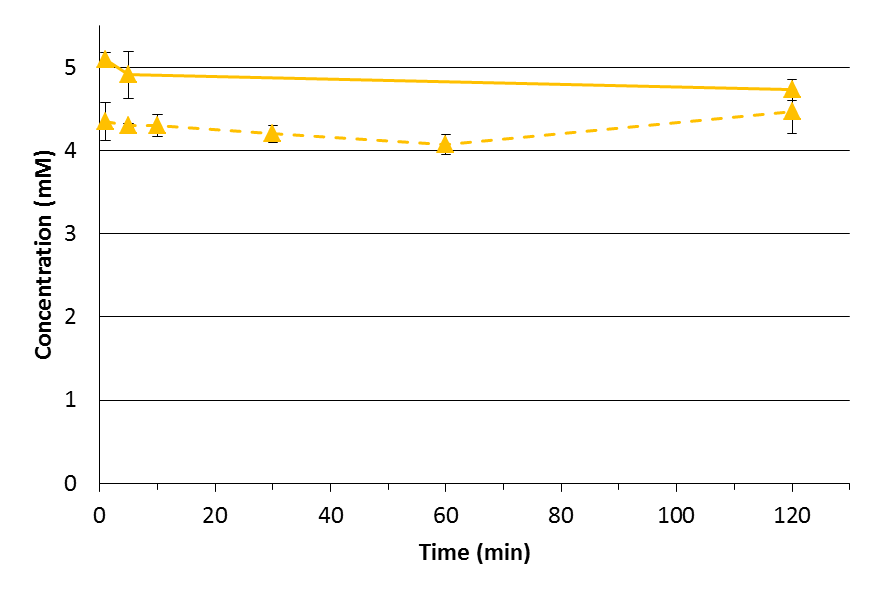


Figure S4. Incubation of 9-hydroxy ethyl nonanoate with 1.0 g_cdw_/L of *E. coli* pCOM10_*alkL* (solid line), and without cells (dashed line). Triangles: 9-hydroxy ethyl nonanoate.


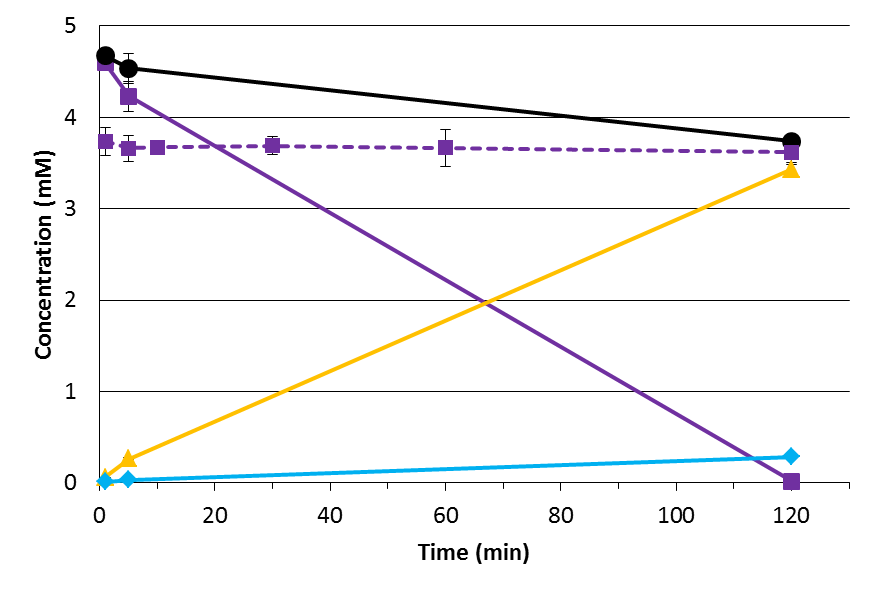


Figure S5. Incubation of 9-oxo methyl nonanoate with 1.0 g_cdw_/L of *E. coli* pCOM10_*alkL* (solid lines), and without cells (dashed line). Squares: 9-oxo methyl nonanoate. Triangles: 9-hydroxy methyl nonanoate. Diamonds: mono-methyl azelate. Circles: sum.

Figure S6. Comparison of the AlkBGTHJ pathway with the AlkBGT (overoxidation) pathway, for ω-oxidation of ethyl esterified fatty acids. The overoxidation *via* AlkBGT is shown in grey.
